# Evaluation of Self-medication for Management of Odontogenic Pain in Iranian Patients

**DOI:** 10.3290/j.ohpd.b1074601

**Published:** 2021-03-17

**Authors:** Nader Navabi, Mina Rakhshanifard, Sepehr Pourmonajemzadeh, Sahand Samieirad, Maryam Alsadat Hashemipour

**Affiliations:** a Associate Professor, Department of Oral Medicine, Faculty of Dentistry, Kerman University of Medical Sciences, Kerman, Iran; Oral and Dental Diseases Research Center, Kerman University of Medical Science, Kerman, Iran. Study design.; b Dentist, Kerman University of Medical Sciences, Kerman, Iran; member of Kerman Dental and Oral Diseases Research Center. Literature search, performed study.; c Dentist, Kerman University of Medical Sciences, Kerman, Iran; member of Kerman Dental and Oral Diseases Research Center. Statistical evaluation.; d Assistant Professor, Oral and Maxillofacial Diseases Research Center, Mashhad University of Medical Sciences, Mashhad, Iran. Idea.; e Professor, Department of Oral Medicine, Faculty of Dentistry, Kerman University of Medical Sciences, Kerman, Iran; Neuroscience Research Center, Institute of Neuropharmacology, Kerman University of Medical Science, Kerman, Iran. Idea, wrote the manuscript.

**Keywords:** analgesic, dentistry, narcotic, odontogenic pain, self-medication

## Abstract

**Purpose:**

Analgesics (painkillers) are one of the most widely used medications to reduce and control pain. The objective of this study was to investigate the self-medication with analgesics (narcotic or non-narcotic) in controlling odontogenic pain in patients visiting dental offices, dental clinics, and the dental school of Kerman.

**Materials and Methods:**

This was a descriptive-analytic study, conducted in 2018. The study sample included patients referring to dental offices, dental clinics and the dental school of Kerman. After obtaining informed consent, a questionnaire consisting of demographic data and questions regarding the consumption of different types of analgesics for relieving and controlling odontogenic pain and their impact on patients was given by the researcher to the respondents. The patients were asked to complete and return the forms. The questionnaire consisted of three categories of questions, including demographic data, pain characteristics (severity, aggravating factors, relieving factors, etc) and the drug used to relieve the pain. Pain severity was measured using a visual analogue scale (VAS). Mann-Whitney and chi-squared tests were used for statistical analysis in SPSS.

**Results:**

This study included 230 males and 351 females (male:female ratio = 0.66) in the age range of 18 to 71 years old (38.21 ± 7.45). 2.6% of respondents were illiterate and 11.3% of respondents were unemployed. The mean value of pain intensity was 6.21 ± 1.11 on a scale of 1 to 10. The types of drugs used for pain relief included 71.8% analgesics, 12.1% complementary medicines and 16.1% antibiotics. The most commonly used medication was NSAIDS, followed by acetaminophen codeine. In this study, the fourth most common medication consumed by patients as an analgesic was amoxicillin. Moreover, it showed that 44.3% (257 individuals) of study participants had used analgesics as self-medication to relieve odontogenic pain, of which 46.08% were males (N = 107) and 42.68% were females (N = 150). The gender of respondents, level of education, and occupation were significantly associated with the consumption of opioid drugs (p = 0.023, p = 0.041, p = 0.011, respectively). Consumption of opioid medications was not statistically significantly correlated with pain intensity (p = 0.115).

**Conclusion:**

The factors affecting the appropriate use of medications are social, economic, cultural, and flaws in the health-care system of a society. This study showed that the medications used to reduce pain included analgesics, traditional drugs, and antibiotics. The rate of self-medication was higher among men and among those having a higher level of education.

Supplementary Material

**Table:** Questionnaire

**Gender:** Male Female **Age (years):** **Place of visit:** Dental School Dental clinic Dental office **Occupation:** Employed Unemployed **Degree completed:** High school Pre-university Colleague Bachelor or master degree or above **How many times have you been to the dentist in the last year?** I haven’t had a visit 2 times More than 2 times **What was the reason for your visit?** Oral examination Dental restoration Dental extraction Root canal therapy Dentures Oral prophylaxis Orthodontic Radiographic Scaling of teeth Other **When did your toothache start?** **What would you do if you had to score from zero to ten?** **What is the nature of your pain?** Stabbing Throbbing Sharp Dull **If the pain is radiating, to which regions does it radiate?** Ear Eye Head Jaw **Do eating and drinking hot foods and beverages aggravate pain?** Yes No I don’t know **Do eating and drinking cold foods and beverages aggravate pain?** Yes No I don’t know **Do eating and drinking sweet foods and beverages aggravate pain?** Yes No I don’t know **Does chewing food aggravate pain?** Yes No I don’t know **Does the pain aggravate when you are in a supine position?** Yes No I don’t know **Did you feel pain at night?** Yes No **I don’t know** Has pain caused you insomnia? Yes No I don’t know **Has pain interfered with your daily activities?** Yes No I don’t know **Show the location of your toothache on the figure.** 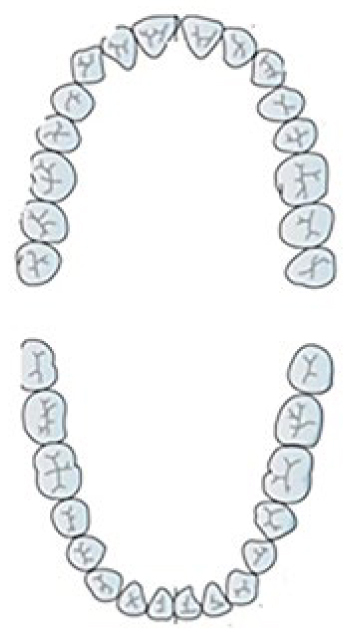 **Have you used medication to relieve pain?** Yes No If so, write down the name of each drug (narcotic or non-narcotic) and how effective it is on the severity of your pain on the specified lines. **No 1: Name of drug:** **Has it been effective in reducing your pain (up to 20 minutes after taking it)?** Yes Severity of pain before use: 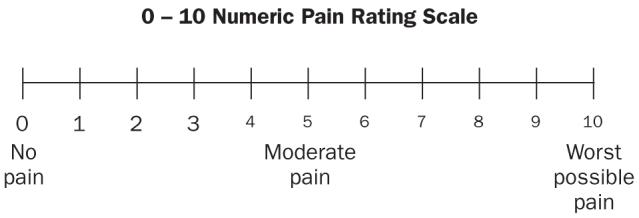 No Severity of pain before use: 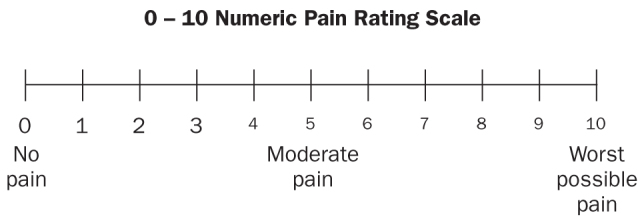 **No 2: Name of drug:** **Has it been effective in reducing your pain (up to 20 minutes after taking it)?** Yes Severity of pain before use: 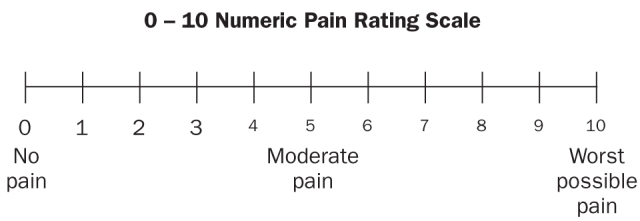 No Severity of pain before use: 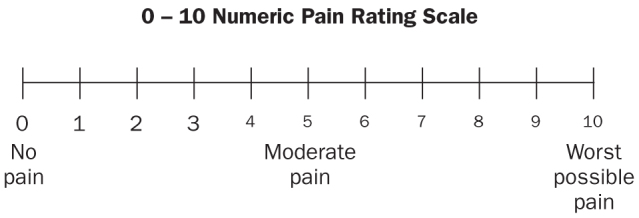 **No 3: Name of drug:** **Has it been effective in reducing your pain (up to 20 minutes after taking it)?** Yes Severity of pain before use: 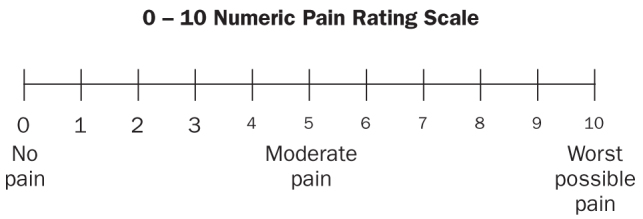 No Severity of pain before use: 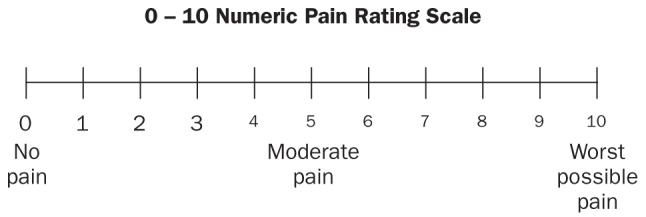 **No 4: Name of drug:** **Has it been effective in reducing your pain (up to 20 minutes after taking it)?** Yes Severity of pain before use: 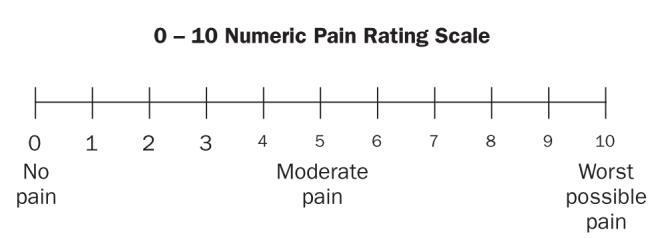 No Severity of pain before use: 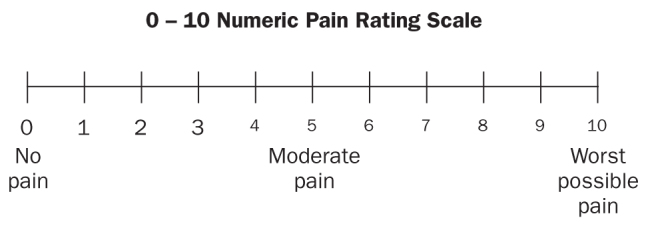

Analgesics, or painkillers, are one of the most widely used medications to control and reduce pain. Analgesics affect the central and peripheral nervous systems through complicated mechanisms.^15,20^ Acetaminophen is one of the most important and most commonly used analgesics, having an antipyretic effect in addition to its analgesic properties. Another important category of painkillers are opioids, the chief of which is morphine and other derivatives of opium. Analgesics are widely used throughout the world. Often, relief is sought for one of the following situations: emotional or chronic stress, hypertension, seizure control, as an adjunct to anesthesia and boosting state of analgesia, analgesia analysis and the control of common pains.^15,20,31,42^

Antipyretic analgesics are a group of drugs that relieve mild to moderate the pain of headache, muscle pain, joint pain, etc and also reduce fever. Some of these medications help to eliminate dysmenorrhea and some are used as anti-inflammatory agents. Antipyretic drugs can be grouped into three general categories on the basis of their mechanisms of action. These include corticosteroids, aspirin and the other NSAIDs (non-steroid anti-inflammatory drugs), and acetaminophen. Each exerts its effect at different points in the febrile response pathway.^9,22^

In the late 2013, a group was set up to participate in an orofacial pain workshop in Montreal, Canada, and all agreed that the use of opioid painkillers by dentists for acute and chronic oral pain had not been properly investigated in that country.^16,22^ Existing literature suggested that the consumption of opioid analgesics for controlling acute pain varies from country to country.^16^ In the UK in 2001, all prescriptions involving analgesics prescribed by dentists were further evaluated, showing the most commonly prescribed analgesic to be ibuprofen (70%).^12^

Drug abuse is a global dilemma and self-administered consumption of medications is increasing worldwide. According to a study by the National Drug Administration in the US in 2003, self-medication with analgesics in individuals aged between 18–25 had increased from 22.1% to 32.2%.^18^

Researchers have shown that on average, 20 doses of opioid analgesics, most likely hydrocodone and oxycodone, are prescribed for patients post-operatively, and most oral and maxillofacial surgeons do not expect patients to use all of the prescribed medications. The remaining opioid analgesics could be an important source for drug misuse.^3,11,19,23,38^

Studies have shown that patients are not sufficiently aware of the proper use of opioid and non-opioid analgesics or their efficacy for pain management.^11,23,28,38^ However, evidence suggested that only a subset of patients would consider opioid medications as efficient over months and years.^17^

The aim of this study was to evaluate the rate of self-administered medication for the management of odontogenic pain in patients that attended private dental offices, dental clinics and the dental school of Kerman in 2018.

## Materials and Methods

This study was designed as a descriptive analytic and cross-sectional study. It was approved by the Institutional Human Research and Ethics Committee of Kerman University of Medical Sciences, Kerman, Iran (ethical approval code EC/94-51/KNRC), and included patients attending the private dental offices, dental clinics and the dental school of Kerman, who completed a questionnaire before having the dental procedure done. The objective of this study was explained individually to each patient, and their participation was voluntary. The patients were assured that all of their information would be kept confidential and would be presented merely as a report. After obtaining patients’ consent, they were given the questionnaire (see supplementary material, below). It consisted of demographic items and questions regarding the consumption of different types of analgesics for controlling odontogenic pain and their efficacy. The patients were asked to complete and return the forms. This study evaluated the data gathered from patients that had referred to the corresponding dental clinics and dental school within 12 months (2018).

In order to achieve validity, questionnaires were provided to 5 experts from Kerman Dental School and they were asked to comment on each question with regard to the options that were as follow: very appropriate, appropriate, no idea, inappropriate and very inappropriate. After collecting, evaluating, and discussing the opinions and the comments of experts about the level and comprehensibility of questions, as well as reviewing the literature, the content validity of questions was deemed acceptable.

The questionnaire’s reliability coefficient was determined by distributing it to 10 patients for 10 days, and they were asked to answer the questions. The reliability coefficient of the questionnaire was determined using Cronbach’s alpha (α = 0.75), which was derived based on statistical consulting at the end of the study. Severity of pain was measured using a Visual Analogue Scale (VAS). The questionnaires consisted of three categories of items, including demographic data, pain characteristics (severity, aggravating factors and relieving factors) and the medication used for pain control. Mann-Whitney, t-test and chi-squared test were used. Statistical analysis was performed using SPSS v 22 (IBM; Armonk, NY, USA).

## Results

In this study, 592 questionnaires were distributed, of which 581 were evaluated (response rate: 98.1%). 351 females and 230 males (male:female ratio = 0.66) responded; their ages ranged from 18 to 71 years old (38.21 ± 7.45). Only 2.6% were illiterate and 11.3% of respondents were unemployed. Table 1 shows the demographic characteristics of the study participants.

**Table 1:** Demographic characteristics of the participants

Demographic variables		No	%
Gender	Male	230	39.58
	Female	351	60.42
Age (years)	35>	385	66.27
	35<	196	33.73
Location of visit	Dental school	45	7.74
	Clinics	415	71.42
	Dental office	121	20.82
Education	High school	45	7.74
	Pre-university	215	37
	College	74	12.73
	Bachelor	175	30.12
	Master degree or above	72	12.39
Occupational status	Employed	515	88.65
	Unemployed	66	11.35

This study showed that 31.8% (n = 185) of participants had not visited a dentist during the last year, 37.2% (n = 220) had only one dental visit, 24.9% (n = 145) had two dental visits and 5.4% (31 patients) had more than two dental visits. The most common reasons for attendance were oral examination, tooth restorations, and endodontic treatment (Table 2).

**Table 2:** Reason for dental visit

Reason for visit	Female		Male	
	No	%	No	%
Oral examination	245	69.80	154	66.95
Restoration	267	76.06	147	63.91
Extraction	123	35.04	134	58.26
Endodontic treatment	201	57.26	123	53.47
Denture	68	19.37	56	24.34
Oral prophylaxis	134	38.17	101	43.91
Radiograph	134	38.17	87	37.82
Orthodontics	31	8.83	22	9.56

334 participants had been suffering from odontogenic pain for less than 2 weeks and the rest for more than two weeks. The reported mean value of pain was 6.21 ± 1.11 (VAS).

Table 3 shows the characteristics of pain of respondents. 513 participants reported that eating and drinking hot foods and beverages aggravated their pain. Also, 494 patients had nocturnal pain.

**Table 3:** Characteristics of odontogenic pain based on gender

Pain Characteristics		Female		Male	
		No	%	No	%
What is the characteristic of your pain?	Throbbing	245	72.36	125	54.34
	Sharp	241	68.66	123	53.47
	Dull	54	15.38	42	18.26
	Stabbing	61	17.37	85	36.95
If the pain is radiating, to which regions does it radiate?	Ear	271	77.20	112	48.69
	Eye	145	41.31	42	18.26
	Head	112	31.90	145	63.04
	Jaw	41	11.68	42	18.26
Do eating and drinking hot foods and beverages aggravate pain?	Yes	301	85.75	212	92.17
	No	50	14.24	18	7.82
Do eating and drinking cold foods and beverages aggravate pain?	Yes	322	91.73	218	94.78
	No	29	8.26	12	5.21
Do eating and drinking sweet foods and beverages aggravate pain?	Yes	302	86.03	195	84.78
	No	49	13.96	35	15.21
Does chewing food aggravate pain?	Yes	300	85.47	185	80.43
	No	51	14.52	45	19.56
Does the pain increase when you are in a supine position?	Yes	321	91.45	210	91.30
	No	30	8.54	20	8.69
Did you feel pain at night?	Yes	310	88.31	184	80
	No	41	11.68	46	20
Has pain caused you insomnia?	Yes	289	82.33	178	77.39
	No	62	17.66	52	22.60
Has pain interfered with your daily activities?	Yes	325	92.59	191	83.04
	No	26	7.40	39	16.95

This study showed that the most common complaint of the participants was the pain felt in the region of posterior mandibular teeth, followed by the maxillary posterior teeth, maxillary premolar area, mandibular premolar area and then the maxillary and mandibular anterior regions. The medications used to relive pain included analgesics (71.8%), traditional drugs (12.1%) and antibiotics (16.1%) (Table 4). It is noteworthy that 33.3% of participants taking antibiotics did not know that they were not analgesics.

**Table 4:** Distribution of drugs consumed based on drug category and gender

Drug category	Female		Male	
	No	%	No	%
NSAIDS	271	77.20	185	80.43
Acetaminophen	245	69.80	45	63.04
Antibiotics	189	53.84	121	52.60
Narcotic drugs	8	2.27	46	20
Home remedies*	102	29.05	81	35.21
Herbal drugs**	84	23.93	58	25.21

*Home remedies include cloves, hollyhock, pomegranate juice, wheat flour. **Herbal remedies include, herbal mouthwashes.

The most commonly used medication was NSAIDS, followed by acetaminophen. This study showed that amoxicillin was the fourth most common medication consumed by patients for pain relief (Table 5).

**Table 5:** The 10 most commonly used medications reported by patients and their impact on pain

Name of drug	Impact of drug on pain				p-value
	Effective		Not effective		
	No	%	No	%	
Ibuprofen	162	79.80	41	20.19	*0.001
Gelofen	81	77.14	24	22.85	*0.001
Mefenamic acid	45	67.16	22	32.83	*0.001
Acetaminophen	115	65.34	61	34.65	*0.001
Acetaminophen codeine	181	84.57	33	15.42	*0.001
Indomethacin	51	62.96	30	37.03	*0.001
Amoxicillin	85	54.48	71	45.51	0.07
Myrtix topical gel	21	28	54	72	0.21
Penicillin	105	68.18	49	31.81	*0.001
Narcotids	36	66.7	18	33.3	*0.001

*p<0.05 is statistically significant.

This study showed that 44.3% (257 people) of participants self-medicated to relieve toothache, of which 46.08% were males (107 men) and 42.68% were females (150 women). Although the rate of self-medication was higher in men, this was not statistically significant (p = 0.12). This is also inferred from the results that younger individuals tended to self-medicate more than the elderly; this difference was statistically significant (p = 0.03).

A positive correlation was found between the level of education and self-medication (p = 0.01): the rate of self-medication was higher among well-educated participants. However, occupation/employment status was not statistically significantly correlated with self-medication (p = 0.09). Furthermore, no statistically significant relationship was found between the consumption of medications and dental care (p = 0.14).

In studying the impacts of sociological variables such as age, gender, education, occupation and the intensity of pain on the rate of consumption of opioid analgesics, it was found that gender, educational level and occupation were statistically significantly related to the consumption of these medications (p = 0.023, p = 0.011, p = 0.041, respectively). Male respondents were more likely to use opioid drugs than were females, the unemployed more than the employed, and uneducated participants more than the well-educated tended to make use of opioid analgesics. The rate of consumption of opioid analgesics was not statistically significantly related to the severity of pain (p = 0.115) (Table 6).

**Table 6:** The impact of demographic variables on consumption of opioid analgesics in patients

Demographic characteristics		No	%	OR (95% CI)	Adjusted odds	p-value
Gender	Male	230	39.58	0.425 (0.345–0.871)	1.123 (0.842–2.341)	*0.011
	Female	351	60.42			
Age	<35	385	66.27	0.541 (0.321–0.912)	1.123 (0.982–2.246)	*0.018
	>35	196	33.73			
Education	Undergraduate	260	44.8	0.412 (0.389–0.732)	1.107 (0.762–2.579)	*0.041
	College or higher	321	55.2			
Occupational status	Employed	515	88.65	0.321 (0.234–0.621)	1.213 (0.980–1.967)	*0.023
	Unemployed	66	11.35			
Mean value of pain	5>	405	69.7	0.721 (0.567–1.541)	1.045 (0.871–1.543)	0.115
	>5	176	30.3			

*p<0.05 is statistically significant; CI: confidence interval; OR: odds ratio.

## Discussion

Using the appropriate medication appropriately is paramount in curing a disease and achieving the desired therapeutic outcomes. Medical experts believe that the correct use of medication in most cases will improve the patient’s condition. On the other hand, recent scientific and industrial advancements in the fields of medicine and pharmacology have made all types of medications readily accessible for patients. If the availability of these drugs is not accompanied by a specific programme, many problems can arise, such as drug abuse and misuse.^50^ Inferring from the content of different studies on substance use and abuse, it is problem affecting aspects of life (eg, cultural, social, religious) in addition to public health. The correct and rational consumption of medications is one of the goals and priorities of the World Health Organization.^18^

Self-administered consumption of medications by patients can lead to drug resistance. This is facilitated by inadequate instructions from health care professionals on correct consumption of medicines and doses, making it a risk-fraught issue. The use of antibiotics in lower doses, and excessive intake of NSAIDs can lead to antibiotic-resistant strains of pathogens, liver damage, and digestive problems.^46^

One of the most important causes of drug-resistant bacteria is the overuse of antibiotics.^35^ Globally, bacterial resistance to antibiotics is one of the greatest dangers to human health in the modern age.^46^ In 2007, the World Health Organization described drug resistance to antibiotics as a “major global threat”.^35^ The WHO reported increasing drug resistance throughout the world by examining statistics from nine countries. The United Nations-affiliated organisation released a report on April 5, 2007, stating that the world had entered a “post-antibiotic” period, a period where simple infections that had been curable for many years were now deadly.^35^

Iran is one of the countries that has over-prescribed these drugs; antibiotic use in this country is approximately equal to its total use in Europe.^15^ Accordingly, antibiotic use in Iran is five times that of the global rate.^15^

Self-medication and inappropriate use of drugs can lead to multiple complications such as drug dependence, excessive and prolonged consumption of drugs, delay in the procedure of treatment of a serious disease, the concealment of symptoms of a severe illness and possible drug interactions,^40^ as well as causing mortality, imposition of extra costs on the pharmaceutical budget of the government’s insurance companies and population of a society.^18^ Moreover, in developing countries, self-administered medication can lead to the formation of inappropriate patterns of drug consumption.^36^

This study examined self-medication with analgesics (opiods). Regarding the importance of this study, Iran is a country that has a very high rate of medication consumption, and over the past decade, drug use has increased uncontrollably.^4,18,26,36,40^ The procurement and production of pharmaceutical products are the responsibility of the primary health-care program, but due to inappropriate prescription of drugs and self-medication, the community of patients is faced with the difficulty of providing their own medications.^4^

This study showed that the medications used to reduce pain included analgesics (71.8%), complementary drugs (12.1%) and antibiotics (16.1%). The most commonly used analgesics are ibuprofen, acetaminophen, combination of acetaminophen and aspirin, and naproxen.^4,8,26,27,43,47^ Pain is commonly the chief complaint of patients attending dental clinics. Analgesics are being widely used arbitrarily by patients, and researchers believe that severe dental pain and difficulty in achieving dental care for patients lead to unnecessary consumption of medications.

A study of self-medication by Anyanechi and Saheeb^6^ on patients suffering from toothache in Nigeria showed that NSAIDS such as aspirin, panadol, and cataflam, as well as antibiotics such as ampiclox and amoxicillin were the most commonly misused drugs. In contrast, the drugs least commonly misused were alcoholic beverages, herbal compounds and active charcoal. The results of their study^6^ were consistent with ours. One of the reasons behind the wide use of antibiotics in our society is their easy accessibility.

The factors facilitating inappropriate use of medications are social, economic, and cultural, as well as flaws in the health-care system of a society. For instance, one of the reasons behind the wide use of antibiotics in our society is their easy accessibility. A study by Jalilian et al^26^ showed that analgesics, antibiotics and medications for common colds were the most frequently purchased drugs from pharmacies in Hamadan, Iran. In the study by Tabiei et al,^49^ the most commonly used drugs in Iran were analgesics. Antibiotics, anti-coagulants and vitamins were the most commonly used drugs in our study, which closely were consistent with the results of other studies.^6,24,29,33,39,52^

Sahebi et al^43^ showed that those who frequented Tabriz (Iran) pharmacies most commonly used analgesics and antibiotics to self-medicate. James et al^27^ stated that analgesics, medications for sore throat and common cold were the most commonly used products for self-medication.^27^ Simon et al,^47^ Kalian et al,^29^ Nayyar et al,^39^ and Beig et al^8^ found similar results.

This study showed that 44.3% of participants self-medicated to relieve toothache. In the study by Nayyar et al,^39^ 33% of participants had practiced self-medication to control and relieve odontogenic pain. Simon et al^47^ also found that 30% of subjects included in the study had self-medicated, which was not consistent with the results of studies in Iran. For example, self-medication was reported to be 83% among students of Yazd city, more than 80% in Ardabil city, 83.7% in Shiraz city and 83.3% among students of Qazvin city. In other countries, the rates of self-medicationwere: Slovenia: 92.3%;^33^ Karachi (Pakistan): 76%^52^ Palestine: 98%;^44^ and in other countries as well it was more than 50%.^24,41,45,48^

A study conducted by Baig et al^8^ revealed a high prevalence (70%) of self-medication for controlling odontogenic pain, which was higher than rates reported in China (32.5%),^21^ India (34.5%),^13^ and Turkey (45%),^14^ but in lower than those of Sudan (73.9%),^7^ Kuwait (92%),^1^ and Rhode Island (USA).^15^ Regional studies conducted in Pakistan showed rates between 46%-76%.^24,52^

The frequency of self-medication increases the frequency of side effects of drugs and the danger of drug interaction, which has a negative effect on the ability to rationally treat a disease in a society.^41,43^ The results of the current study were in accordance with those of a study conducted in Turkey by Ilben et al,^25^ in which the rate of self-medication in men was higher than that of women. Also, Anyanech and Saheeb^6^ showed that men self-medicate more often than women. This difference can be due to the fact that women are more likely to seek treatment than men; thus, fewer females self-medicated. In the study by Simon et al,^47^ women were more likely to self-medicate than men, which was consistent with the results of similar studies.^2,5,7,8,34,51^ According to Baig et al,^8^ this could be due to the lower threshold of pain in women and their fear of dental treatment. Women who participated in these studies were often from low socioeconomic levels and they were mostly unemployed.

In the study by Tabiei et al,^49^ the rate of consumption of medications without a physician’s prescription was the same among men and women. But in Palestine, drug usage was reported more frequent among women (63.4%).^44^ The rates reported for Tabriz city was 52%^34^ and 88.3% in men in Shiraz city.^32^, This difference may be related to the number of samples per group.

Unlike developing countries, prescribed drugs are easily available over the counter (without prescription). There are strict rules in European countries regarding the sale of medications. This could be the main reason why self-medication happened more among the participants of this study.

This study showed that younger individuals tended to self-medicate more than the elderly, and there was a significant relationship in this regard. In the studies by Anyanechi and Saheeb^8^ and Nayyar et al,^39^ self-medication was more common among elderly who were suffering from odontogenic pain; however, Lawan et al^34^ did not report a statistically significant relationship between respondent’s age and self-medication.

In the study by Tabiee et al,^49^ the highest frequency of self-medication was reported among individuals younger than 20 years, but in the study in Shiraz city,^32^ with increasing the age, there was an increase in self-medication, which could be due to cultural differences, lifestyle and socioeconomic factors. Afolabi et al^3^ reported drug use in both young and old people. However, the difference in results could be ascribed to the presence of chronic diseases among the group of patients.

This study found a positive correlation between the level of education and self-medication. In several studies,^8,10,29,45^ there was a positive correlation between education and self-medication, but in the study by Simon et al^47^ such a correlation was not observed.

The study by Anyanechi and Saheeb^6^ showed that the use of analgesics, antibiotics and saline mouthwash was more common among people from upper socioeconomic levels. On the other hand, the use of herbal drugs, hot alcoholic drinks and active charcoal was more common among those with a lower socioeconomic background. This could indicate their beliefs about healthcare as well as that traditional medications have a partial impact on those diseases.

Education has a special role in self-medication: individuals with higher education have higher levels of self-confidence, enabling them to take drugs without the prescription of dentists, while illiterate people recognise drugs based on their color and name. Another parameter measured in the current study was the occupation of participants: there were no obvious correlations between occupation and self-medication. This agrees with results by Lawan et al^10^ in Nigeria. Occupation could be a measure of income, but in this study, there was no correlation between family income and self-medication. Other factors such as the lack of free time and stress can affect the relationship between occupation and self-medication. There are also studies confirming the association between low socio-conomical level and higher rates of self-medication.^2,6,34^

This study showed that there was a statistically significant relationship between self-medication and referral to dental office. In the study by Simon et al,^47^ no statistically significant correlation was found between self-medication and visit to the dentist, but self-medication was directly proportional with the elapsed time since the last dental visit. This correlation can be explained by the fact that at the time of last dental visit, the practitioner gave instructions as a guide for patients to take medications. This finding emphasised that regular dental visits can affect self-medication. This study showed that the gender of respondents, their educational level and respondent’s occupation were significantly associated with the consumption of narcotic drugs.

Male respondents were more likely than females to use these drugs. Unemployed individuals were more likely to utilise these drugs compared to well-educated people. A review of search engines showed that so far, no studies have been conducted on the efficacy of narcotic analgesics on dental pain, therefore, a comparison of results was not possible.

Moore et al^37^ showed that taking 325 mg of acetaminophen and 200 mg of ibuprofen after extraction of third molars was more effective in managing post-operative pain than were opioid analgesics. They also concluded that the combination of ibuprofen and acetaminophen could be more effective than many present formulations. Kissin’s recent article on the use of opioid drugs to treat non-cancer chronic pain showed that these drugs had no statistically significant effects.^30^ A recent Cochrane review compared the effects of narcotic drugs and placebo in the treatment of back pain.^17^ The results of that study showed that these drugs were more effective in short-term treatment than placebo, although there was no evidence of their long-term effect.^17^

Finally, the important points of this study were that medication is relatively commonly used to relieve toothache, opioids are often used to relieve pain, and people also mistakenly use antibiotic drugs as painkillers and.

## Conclusion

Self-medication was more often practiced among males and well-educated individuals. The most commonly used medication was NSAIDS, followed by acetaminophen codeine. The fourth most common medication consumed by patients as an analgesic was amoxicillin.
